# Are infraslow oscillations the missing link between sleep and Alzheimer's?

**DOI:** 10.1002/alz.71525

**Published:** 2026-09-08

**Authors:** Demetrio Grollero, Victoria Gabb, Jonathan Blackman, Amanda Heslegrave, Luisa de Vivo, Elizabeth Coulthard, Michele Bellesi

**Affiliations:** ^1^ Department of Experimental and Clinical Medicine Marche Polytechnic University Ancona Italy; ^2^ School of Pharmacy University of Camerino Camerino Italy; ^3^ Center for Neuroscience University of Camerino Camerino Italy; ^4^ Bristol Medical School University of Bristol Bristol UK; ^5^ Department of Neurodegenerative Diseases University College London London UK; ^6^ School of Biosciences and Veterinary Medicine University of Camerino Camerino Italy

**Keywords:** Alzheimer's disease, EEG biomarkers, glymphatic system, infraslow oscillations, locus coeruleus, sleep spindles

## Abstract

**INTRODUCTION:**

Locus coeruleus (LC) and glymphatic dysfunction have been linked both to Alzheimer's disease (AD) and, recently, to infraslow oscillation (ISO) in sleep spindle (sigma) activity (ISO). Here we hypothesize ISO integrity is a critical link between sleep and AD.

**METHODS:**

We analyzed non‐rapid eye movement sleep electroencephalogram (EEG) data from AD participants and controls, extracting ISO peak amplitude, intrinsic frequency, and bandwidth from the sigma‐power time‐course. We assessed group differences and correlations with plasma biomarkers (Aβ42/40, pTau181, pTau217, neurofilament light chain [NfL], glial fibrillary acidic protein [GFAP]).

**RESULTS:**

ISO peak amplitude was significantly reduced in AD, while intrinsic frequency and bandwidth were preserved. ISO peak amplitude showed a trend‐level association with Aβ42/40, whereas ISO bandwidth was positively associated with NfL, and showed trends with GFAP and lower verbal memory retention.

**DISCUSSION:**

Selective weakening of ISO in AD is consistent with LC dysfunction and impaired glymphatic cycling. ISO may be a novel mechanism and electrophysiological marker linking sleep microarchitecture to AD pathology.

## Highlights

- Infraslow oscillation (ISO) of sleep spindle activity is reduced in Alzheimer's disease.
- ISO peak amplitude is selectively reduced, while frequency and bandwidth are preserved.
- ISO amplitude shows a trend‐level association with plasma Aβ42/40 ratio.
- ISO bandwidth is associated with NfL and shows trends with glial fibrillary acidic protein (GFAP) and memory retention.
- ISO metrics link sleep microarchitecture to LC‐related and glymphatic processes in AD.

## 1 BACKGROUND

Sleep architecture is profoundly altered in Alzheimer's disease (AD), and growing evidence suggests that these disruptions are not merely symptomatic but mechanistically linked to neurodegenerative processes.^1^, ^2^, ^3^ In particular, non‐rapid eye movement (NREM) sleep, during which coordinated oscillatory activity supports memory consolidation, synaptic homeostasis, and glymphatic clearance, markedly deteriorates early in the disease course.^4^, ^5^, ^6^ Among the key electrophysiological markers of NREM sleep, spindle‐related sigma (12–16 Hz) activity has emerged as a sensitive indicator of thalamocortical circuit integrity and cognitive function.^7^, ^8^ Recent work has revealed that NREM sigma power is not constant but manifests infraslow oscillation (ISO), such that sleep spindles temporally cluster at < 0.1 Hz.^9^, ^10^

The neural substrates of these ISO dynamics remain an active area of investigation. Animal and pharmacological studies have proposed that ISO periodicity is driven by rhythmic fluctuations in locus coeruleus (LC) firing, which generate corresponding cycles of noradrenaline (NA) release, modulating cortical arousal and gating sleep spindle expression during NREM sleep.^10^, ^11^, ^12^ Recent evidence further suggests that other neuromodulatory systems may oscillate in concert at infraslow timescales.^13^ While the precise neuromodulatory architecture underlying infraslow sigma modulation in humans remains to be fully established, these observations have led to the proposal that infraslow modulation of sigma activity may reflect underlying noradrenergic cycling during sleep.

Furthermore, animal models suggest that oscillatory fluctuations in noradrenergic activity during NREM sleep modulate pericyte contraction, astrocytic end‐feet geometry, and vascular tone, thereby promoting cerebrospinal fluid (CSF) influx and metabolic waste clearance, including β‐amyloid.^14^, ^15^ However, the extent to which infraslow sigma oscillations index these processes in humans remains uncertain.

The LC is among the earliest sites of tau‐related pathology in AD, and impaired glymphatic function has been proposed as a key contributor to β‐amyloid accumulation.^16^, ^17^, ^18^ In this context, alterations in infraslow sigma modulation could represent a downstream electrophysiological correlate of disrupted arousal regulation, potentially linking LC compromised integrity and reduced glymphatic clearance.

In this study, we investigated the integrity of the ISO of sigma power in individuals with AD compared with age‐matched cognitively unimpaired control participants. We hypothesized that the integrity of the ISO of sigma power in individuals with AD would be reduced compared with age‐matched healthy control participants.

## 2 MATERIALS AND METHODS

### 2.1 Participants

Thirty older adults (age > 50 years) were selected from the Remote Evaluation of Sleep to Enhance Understanding of Early Dementia (RESTED) ‐AD cohort,^19^ a longitudinal observational study of sleep physiology in individuals on the AD spectrum (including mild cognitive impairment [MCI] and AD dementia; *N* = 10) matched with cognitively unimpaired participants (*N* = 20). Demographic and clinical characteristics of the participants are summarized in Table S1. Clinical and behavioral measures were assessed using standardized instruments. Global cognitive function was evaluated with the Montreal Cognitive Assessment (MoCA), depressive symptoms with the Geriatric Depression Scale (GDS), anxiety symptoms with the Generalized Anxiety Disorder scale (GAD‐7), and sleep quality with the Pittsburgh Sleep Quality Index (PSQI). Additional clinical and behavioral measures were collected as part of the RESTED‐AD protocol but are not reported here. All participants met the RESTED‐AD inclusion criteria and provided written informed consent. Details on recruitment, ethics, diagnostic procedures, and multimodal phenotyping have been described previously.^19^

**RESEARCH IN CONTEXT**

1. **Systematic review**: We reviewed the literature using PubMed to identify studies on sleep alterations in Alzheimer's disease (AD), locus coeruleus (LC) dysfunction, glymphatic clearance, and infraslow modulation of sleep spindle activity. Although sleep spindle and slow‐wave abnormalities in AD are well documented, no prior studies have specifically examined infraslow oscillations of sigma power or their association with plasma biomarkers of AD pathology.
2. **Interpretation**: This study provides the first evidence that infraslow oscillation (ISO) amplitude of sleep spindle activity is selectively reduced in AD and shows trend‐level associations with amyloid burden, while ISO bandwidth is associated with markers of neurodegeneration and shows trend‐level associations with memory performance. These findings link sleep microarchitecture to molecular and cognitive features of AD and support a role for LC‐glymphatic dysregulation in disease mechanisms.
3. **Future directions**: Future studies should determine whether ISO alterations assess their relationship with in vivo measures of LC integrity and glymphatic function, and test whether interventions targeting noradrenergic or sleep regulation can restore ISO dynamics and modify AD biomarkers.

### 2.2 Protocol and procedures

RESTED‐AD was a naturalistic, home‐based study designed to capture variability in sleep and behavior under real‐world conditions. Following baseline clinical and neuropsychological assessment, participants underwent an intensive monitoring phase. Sleep was recorded for seven consecutive nights at home using a wireless multichannel portable electroencephalogram (EEG) device (Dreem 2), with recordings self‐initiated at habitual bedtime.

### 2.3 EEG preprocessing

EEG data were band‐pass filtered (0.5–35 Hz), and artifacts were removed using an automated procedure followed by visual inspection. Artifact‐free NREM2 segments of at least 5 min were selected for analysis (see Supplementary Methods for a detailed description).

### 2.4 ISO extraction

Sigma‐band activity (10–16 Hz) was extracted using time–frequency decomposition, and the amplitude envelope was used to quantify ISO (< 0.1 Hz). ISO features (peak amplitude, frequency, and bandwidth) were derived from the power spectrum of the sigma envelope using a model‐based fitting approach (see Supplementary Methods for a detailed description).

### 2.5 Plasma biomarkers

Blood samples were collected, processed, and stored according to standardized procedures. Briefly, samples were centrifuged (10 min at ∼1800 rcf), plasma was aliquoted, and stored at −80°C until analysis. Plasma biomarkers were quantified using the Quanterix HD‐X platform (single‐molecule array [Simoa] technology), including pTau‐217 (ALZpath Simoa pTau‐217 v2), pTau‐181 (Simoa pTau‐181 V2.1 Advantage), and a multiplex panel for Aβ40, Aβ42, glial fibrillary acidic protein (GFAP), and neurofilament light chain (NfL) (Simoa Neurology 4‐Plex E).

### 2.6 Memory evaluation

Memory performance was assessed using a word‐list task as described in Blackman et al.^20^. Participants encoded words in the evening and were tested the following morning. Analyses were restricted to the standard / non‐autobiographical condition. Three measures were derived: recognition sensitivity (*d*′), memory retention, and effective recall. Recognition sensitivity (*d*′) was calculated using signal detection theory (hits, misses, false alarms, correct rejections). Memory retention was defined as the ratio of delayed to immediate recall, and effective recall as correct recalls minus intrusions. Standard corrections were applied, and participants with missing data were excluded.

### 2.7 Statistical analysis

Statistical analyses were performed in MATLAB (v2021) and GraphPad Prism (v10.6.01). Group differences in ISO features were assessed using linear mixed‐effects models with subject included as a random intercept to account for the hierarchical structure of repeated observations. Parameters were estimated using restricted maximum likelihood (REML), and statistical significance of group effects was evaluated using F‐tests with Satterthwaite approximation of degrees of freedom. Results were additionally verified at the subject level by averaging ISO features within participant prior to group comparison using two‐sample t‐tests, following confirmation of normality after log‐transformation. For correlations with plasma biomarkers and cognitive measures, ISO features were averaged across nights to obtain subject‐level estimates. Correlational analyses with plasma biomarkers were structured into two predefined sets. Primary analyses focused on canonical markers of AD pathology (Aβ42/40, pTau181, pTau217), whereas associations with GFAP and NfL were considered exploratory. For each ISO feature, *p*‐values were adjusted across biomarkers within each set using the Benjamini–Hochberg false discovery rate (FDR; *q* = 0.05). Correlational analyses with cognitive measures were considered exploratory, and for each ISO feature, *p*‐values were adjusted across cognitive outcomes (*d*′, retention, effective recall) using the same FDR procedure. Sample sizes varied across analyses due to missing data; *N* is reported for each correlation. All tests were two‐tailed with a significance threshold of α = 0.05.

## 3 RESULTS

### 3.1 Dataset quality description

After quality control, one participant was excluded due to poor EEG data quality. A total of ∼2,050 artefact‐free 5‐minute NREM2 segments (∼170 h of NREM sleep) were retained across 152 nights from 29 participants. Nights with fewer than two valid segments (10.53%) or without a dominant infra‐slow oscillatory peak (7.9%, see the Supplementary Methods section) were excluded, resulting in a final sample of 124 nights (AD: 41 nights from nine participants; controls: 83 nights from 18 participants). No differences were observed between groups in the number of nights included per subject (*p* = 0.95) or in the average number of valid NREM2 segments per night (*p* = 0.21; Figure S1). Sleep architecture quantification is summarized in Table S2.

### 3.2 ISO of the sigma power is reduced in AD

**FIGURE 1:**
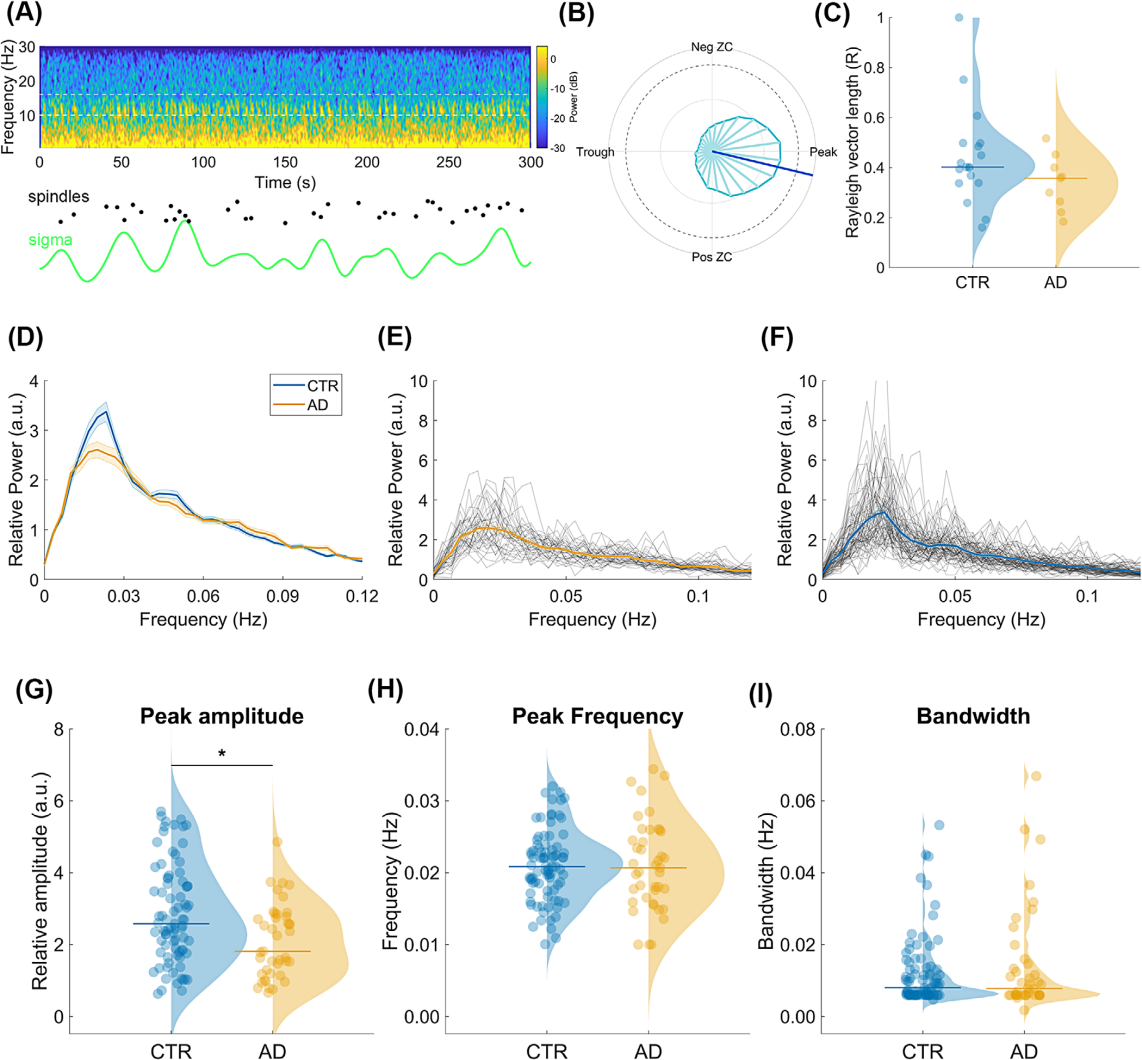
Group differences in ISO spectra and features. (A) Representative example of infraslow sigma modulation during NREM2 sleep. Top: time‐frequency representation of EEG activity (0‐30 Hz) over a 300 s segment; white dotted lines delimit the sigma band. Bottom: corresponding sigma amplitude envelope filtered in the infraslow range (0.01–0.04 Hz, green), illustrating the rhythmic infraslow modulation of sigma activity. Black dots indicate detected spindle events. (B) Circular distribution of spindle occurrence across the phase of the sigma ISO, pooled across all participants (*N* = 27; N spindles = 10,340). The gray dotted line indicates a uniform 10% occurrence probability per bin; the blue vector denotes the preferred phase. Spindles clustered consistently around the positive ISO peak at the pooled (μ = −0.23 rad, *R* = 0.36; Rayleigh *p* < 0.001), night (*N* = 120; μ = −0.23 rad, *R* = 0.89; *p* < 0.001), and subject level (*N* = 27; μ = −0.27 rad, R = 0.93; *p* < 0.001), indicating robust phase locking across nights and participants. (C) Subject‐level spindle phase clustering by group. Rayleigh vector length (R) reflects the strength of phase concentration. Colored dots represent individual participants; horizontal lines denote group mean ± SEM. No significant group difference in clustering strength was observed (CTR: *R* = 0.44 ± 0.046; AD: *R* = 0.34 ± 0.036; *p* = 0.17). (D–F) Group‐average night‐level spectra for CTR (blue) and AD (yellow) are shown as mean ± SEM across nights. Individual night‐level spectra (thin grey lines) for AD (E) and CTR (F) are also presented, with the corresponding group means ± SEM overlaid. Spectra are expressed in relative units (a.u.) with respect to the 0.006–0.1 Hz frequency band. For visualization only, ultra‐low‐frequency peaks (< 0.006 Hz), attributable to residual baseline offsets and outside the predefined infra‐slow range, were masked prior to averaging. (G–I) Distribution of ISO features in CTR (blue) and AD (yellow). Panels display peak amplitude (G), peak frequency (H), and bandwidth (I) of the infraslow modulation of sigma power. Individual dots represent single nights, half‐violins depict kernel density estimates, and horizontal lines indicate the median value within each group. *p < 0.05. AD, Alzheimer's disease; CTR, control; EEG, electroencephalograph; ISO, infraslow oscillation; NREM, non‐rapid eye movement; SEM, standard error of the mean.

The ISO was extracted from the EEG sigma power time course. Consistent with prior research,^9^, ^10^, ^11^ the ISO showed a clear dependence on spindle activity, with detected spindles clustering to a specific phase of the ISO cycle (Figure 1A). This temporal organization was preserved across both slow and fast spindles, and did not differ between AD and control participants (Figure S2, Figure 1B), confirming that the spectral ISO measure captures a physiologically meaningful and robust oscillatory process. We then quantified the modulation of ISO by extracting three features from each participant's ISO spectrum of the sigma power time course: (i) the ISO peak amplitude, reflecting the strength of the rhythmic oscillation; (ii) the ISO intrinsic frequency, representing the dominant oscillatory rate within the infraslow range; and (iii) the ISO bandwidth, capturing the spread of spectral power around the peak frequency. Across groups, a clear reduction emerged in the ISO peak amplitude in AD patients compared with cognitively unimpaired controls (‐26.8 ± 7.46%, *p* = 0.024, Figure 1), indicating a marked weakening of the ISO amplitude of sigma activity. In contrast, neither the ISO intrinsic frequency (*p* = 0.95) nor the ISO bandwidth (*p* = 0.77) differed significantly between groups (Figure 1), suggesting that while the rhythmic structure and frequency of the oscillation are preserved, its overall strength is selectively diminished in AD. This pattern was consistent when analyses were conducted at the subject level (Figure S3). In addition, broad EEG spectrum analysis did not reveal significant reductions in delta or spindle power in AD relative to controls (Figure 4), as observed in prior studies.^21^ Moreover, none of ISO features correlated with the extent of delta in patients and controls (Amplitude: *r* = −0.06, *p* = 0.8; Frequency: *r* = −0.04, *p* = 0.85; Bandwidth: *r* = 0.18, *p* = 0.4, not shown).

### 3.3 ISO features correlate with plasma biomarkers of disease

**FIGURE 2:**
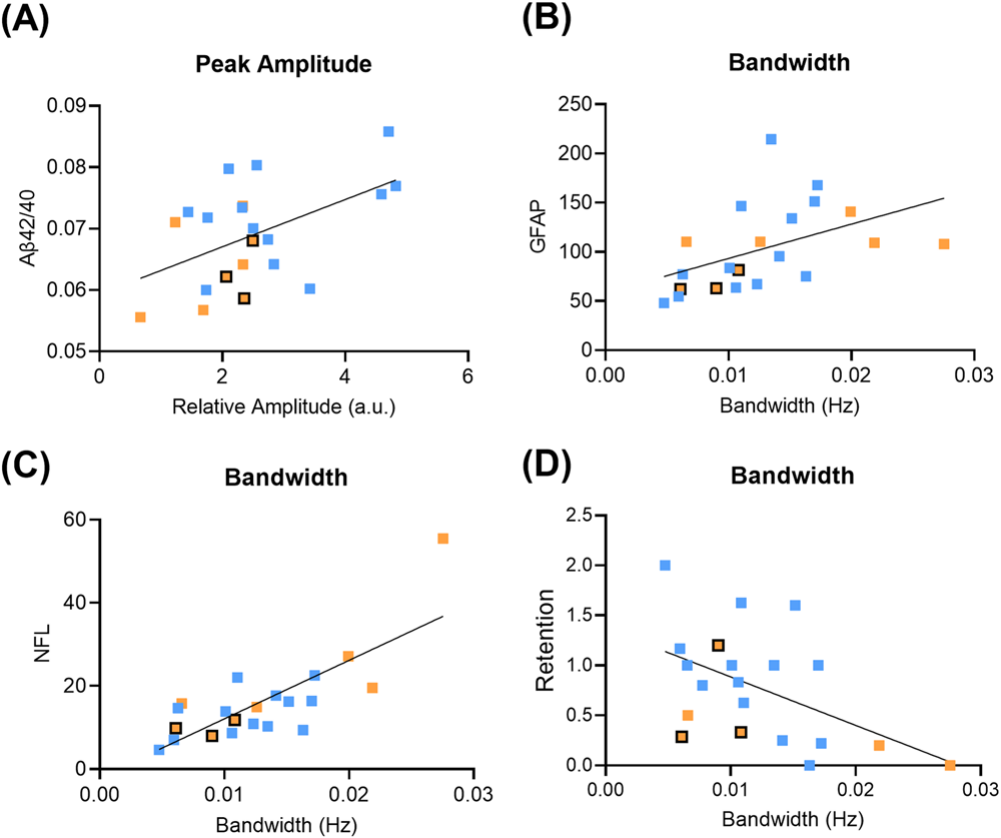
Associations between ISO features, plasma biomarkers, and memory performance. (A) Correlation between ISO peak amplitude and Aß42/40 ratio. (B‐C) Correlation between ISO bandwidth with GFAP (B) and NfL (C) concentrations. (D) Correlation between ISO bandwidth and memory retention. For all panels, yellow squares represent AD (MCI indicated by a black border), while the blue ones are CTR. GFAP, glial fibrillary acidic protein; ISO, infraslow oscillation; MCI, mild cognitive impairment; NfL, neurofilament light chain.

We next assessed whether alterations in ISO features were related to plasma biomarkers of AD pathology and neurodegeneration. Associations with canonical markers of AD pathology (Aβ42/40, pTau181, pTau217) were considered primary, whereas associations with GFAP and NfL were considered exploratory. ISO peak amplitude showed a positive association with the Aβ42/40 ratio (Figure 2A), such that individuals with greater infraslow oscillatory strength tended to exhibit more favorable amyloid profiles (adjusted *p* = 0.057). pTau181 and pTau217 were not significantly associated with ISO peak amplitude (Table 1).

**TABLE 1 alz71525-tbl-0001:** ISO features correlations with plasma biomarkers.

ISO feature	Aβ42/40 (*n* = 21, 8 AD)	pTau 181 (*n* = 24, 9 AD)	pTau 217 (*n* = 20, 7 AD)	GFAP (*n* = 21, 8 AD)	NfL (*N* = 21, 8 AD)
Amplitude	*R* = 0.5, *p* = 0.019, *q* = 0.057	*R* = 0.2, *p* = 0.37, *q* = 0.56	*R* = 0.3, *p* = 0.16, *q* = 0.24	*R* = 0.15, *p* = 0.5, *q* = 0.93	*R* = −0.02, *p* = 0.93, *q* = 0.93
Frequency	*R* = −0.01, *p* = 0.97, *q* = 0.97	*R* = 0.09, *p* = 0.7, *q* = 0.97	*R* = 0.1, *p* = 0.6, *q* = 0.97	*R* = −0.3, *p* = 0.16, *q* = 0.24	*R* = −0.3, *p* = 0.13, *q* = 0.2
Bandwidth	*R* = −0.04, *p* = 0.85, *q* = 0.85	*R* = 0.4, *p* = 0.09, *q* = 0.13	*R* = 0.4, *p* = 0.07, *q* = 0.13	*R* = 0.5, *p* = 0.03, *q* = 0.09	*R* = 0.8, *p* < 0.0001, *q* = 0.0003

*Note*: Pearson correlation coefficients (*R*), uncorrected *p*‐values, FDR‐adjusted *q*‐values, and number of participants are reported.

Abbreviations: FDR, false discovery rate; GFAP, glial fibrillary acidic protein; ISO, infraslow oscillation; NfL, neurofilament light chain.

In addition, ISO bandwidth showed a positive association with plasma NfL levels and a trend with GFAP (NfL: adjusted *p* = 0.0003; GFAP: adjusted *p* = 0.09) across AD and control groups (Table 1). Participants with broader infraslow spectral bandwidth tended to exhibit higher concentrations of these biomarkers (Figure 2B–C), suggesting that greater dispersion in infraslow spectral features may relate to glial and neurodegenerative processes. No significant associations were found for the ISO intrinsic frequency (Table 1).

### 3.4 ISO bandwidth relates to memory performance

Finally, we examined the relationship between ISO features and memory performance on a word list task. ISO bandwidth showed a negative association with word retention (*R* = −0.49, *p* = 0.03, Figure 2D), indicating that individuals with broader infraslow bandwidth exhibited poorer memory performance (Table 2). However, this association did not survive correction for multiple comparisons (adjusted *p* = 0.075) and should therefore be interpreted cautiously. Other ISO features were not significantly related to any of the memory task parameters (Table 2).

**TABLE 2 alz71525-tbl-0002:** ISO Features correlations with memory metrics.

ISO feature	*d*prime	Retention	Recall
Amplitude	*R* = 0.1, *p* = 0.65, *q* = 0.85	*R* = 0.16, *p* = 0.5, *q* = 0.70	*R* = 0.3, *p* = 0.2, *q* = 0.3
Frequency	*R* = −0.04, *p* = 0.85, *q* = 0.85	*R* = 0.09, *p* = 0.7, *q *= 0.70	*R* = 0.2, *p* = 0.3, *q* = 0.3
Bandwidth	*R* = −0.43, *p* = 0.06, *q* = 0.17	*R *= −0.5, *p* = 0.03, *q* = 0.075	*R* = −0.24, *p* = 0.3, *q* = 0.3

*Note*: Pearson correlation coefficients (*R*), uncorrected *p*‐values, and FDR‐adjusted *q*‐values are reported. Data are derived from 20 participants (6 AD).

Abbreviations: FDR, false discovery rate; ISO, infraslow oscillation.

## 4 DISCUSSION

This study examines whether alterations in ISO dynamics are detectable in human sleep and relate to molecular markers of AD pathology.[Table alz71525-tbl-0001], [Table alz71525-tbl-0002] By quantifying ISO features from overnight EEG, we demonstrate a selective reduction in ISO peak amplitude in people with clinically‐diagnosed AD, while intrinsic frequency and bandwidth remain preserved. This pattern reveals a specific weakening of the rhythmic modulation that structures sigma activity. Given that infraslow sigma modulation has been linked to LC‐mediated arousal cycling in experimental models, the ISO reduction observed in AD is compatible with altered noradrenergic regulation during sleep, although LC activity was not directly measured here.

Our findings offer a novel perspective by showing that ISO alterations are not only present in AD but are meaningfully related to plasma biomarkers of amyloid dysregulation and neurodegeneration. The positive association between ISO amplitude and Aβ42/40 ratio indicates that preserved infraslow modulation accompanies a more favorable amyloid profile. Although this association did not survive FDR correction (adjusted *p* = 0.057), the effect size was moderate and in the hypothesized direction. Given the limited sample size, this finding should be interpreted cautiously but may represent a biologically meaningful signal warranting further investigation in larger cohorts. In addition, associations between ISO bandwidth and NfL, together with a trend with GFAP, suggest that additional spectral properties may track glial activation and axonal injury. Specifically, broader ISO bandwidth corresponds to a less sharply defined infraslow oscillatory peak and greater dispersion of power across frequencies, a pattern consistent with reduced ISO stability. No significant associations were found for the ISO intrinsic frequency. In parallel, the association between broader ISO bandwidth and poorer word retention provides initial evidence that alterations in infraslow sigma modulation may also relate to sleep‐dependent memory processes impaired in AD.^7^, ^22^ Finally, ISO features did not correlate with delta power, the canonical electrophysiological marker of sleep depth frequently altered in AD.^21^, ^23^ This dissociation suggests that ISO abnormalities are not driven by reduced sleep intensity but instead reflect a distinct regulatory process, consistent with recent observations showing that NREM noradrenergic oscillations are more tightly coupled to light sleep features, such as microarousals, than to slow‐wave sleep per se.^14^

Several limitations should be acknowledged. The study is cross‐sectional, preventing direct inference about temporal ordering between ISO disruption and biomarker changes. LC function and glymphatic activity were not measured directly, and EEG‐derived ISO features represent downstream proxies rather than mechanistic readouts. Sample size and technical factors related to the recording device (including bipolar derivations, limited spatial coverage, and the use of dry electrodes) may influence the estimation of sigma activity and its infraslow modulation, potentially reducing sensitivity to regional variability and subtler associations. Such variability may affect the estimation of infraslow spectral bandwidth, potentially contributing to increased dispersion in this metric. However, the multi‐night, home‐based design enhances ecological validity and supports more reliable estimation of infraslow dynamics.

Despite these limitations, the present findings have clear implications. They suggest that reduced ISO amplitude may represent an electrophysiological correlate of broader regulatory disruption, potentially involving LC‐noradrenergic and glymphatic systems and offering a testable framework for understanding how sleep microarchitecture contributes to AD progression. Specific next steps will include: (1) longitudinal evaluation to determine whether ISO amplitude predicts cognitive decline or biomarker trajectories; (2) integration with LC imaging (neuromelanin magnetic resonance imaging [MRI] or noradrenergic positron emission tomography [PET]) to directly link ISO alterations to LC integrity; and (3) intervention studies targeting noradrenergic tone or arousal stability to assess whether ISO amplitude can be restored and whether such restoration enhances glymphatic function or reduces pathological protein burden.

In conclusion, this work identifies weakened infraslow modulation of sigma activity as a potential electrophysiological signature of AD‐related network disruption and potential clearance impairment. Establishing ISO features as accessible, biologically grounded biomarkers may open new avenues for early detection and mechanistically guided therapeutic strategies.

## AUTHOR CONTRIBUTIONS

Conceptualization: Michele Bellesi, Elizabeth Coulthard, Jonathan Blackman. Methodology: Elizabeth Coulthard, Jonathan Blackman, Amanda Heslegrave, Demetrio Grollero, Michele Bellesi. Investigation: Victoria Gabb, Jonathan Blackman, Elizabeth Coulthard. Project administration: Victoria Gabb, Demetrio Grollero, Michele Bellesi. Resources: Victoria Gabb, Jonathan Blackman, Elizabeth Coulthard, Luisa de Vivo, Michele Bellesi. Formal Data analysis: Demetrio Grollero, Michele Bellesi. Data curation and graphics: Demetrio Grollero, Victoria Gabb, Jonathan Blackman. Writing‐original draft: Demetrio Grollero, Michele Bellesi; writing‐review and editing: all authors. Project supervision and funding: Michele Bellesi, Luisa de Vivo, Elizabeth Coulthard, Jonathan Blackman.

## CONFLICT OF INTEREST STATEMENT

The authors declare no conflicts of interest. Author disclosures are available in the Supporting Information.

## CONSENT STATEMENT

All participants provided written informed consent, and the study was approved.by the Health Research Authority (Yorkshire and the Humber‐Bradford Leeds Research Ethics Committee, reference 21/YH/0177).

## Supporting information




**Supporting Information**: alz71525‐sup‐0001‐SuppMat.docx

**Supporting Information**: alz71525‐sup‐0002‐SuppMat.pdf

## Data Availability

Data and informed consent forms are available upon request.
