# Supplementary material for: Are infraslow oscillations the missing link between sleep and Alzheimer's?
Source: Alzheimers Dement. 2026 Sep 8;22(9):e71525. doi: 10.1002/alz.71525 (PMC13551593; doi:10.1002/alz.71525)
Supplement: Supplementary file 2 [file ALZ-22-e71525-s002.docx]

**SUPPLEMENTARY MATERIAL**

**Are infraslow oscillations the missing link between sleep and Alzheimer’s?**

Demetrio Grollero^1,2,3^, Victoria Gabb^4^, Jonathan Blackman^4^, Amanda Heslegrave^5^, Luisa de Vivo^2,3^, Elizabeth Coulthard^4^, Michele Bellesi^3,6^

1. Department of Experimental and Clinical Medicine. Marche Polytechnic University, Ancona, IT
2. School of Pharmacy, University of Camerino, Camerino, IT
3. Center for Neuroscience, University of Camerino, Camerino, IT
4. Bristol Medical School, University of Bristol, Bristol, UK
5. Department of Neurodegenerative Diseases, University College London, London, UK
6. School of Biosciences and Veterinary Medicine, University of Camerino, Camerino, IT

Correspondence to:

Prof. Michele Bellesi

School of Biosciences and Veterinary Medicine

University of Camerino

60032, Camerino (MC), Italy

[michele.bellesi@unicam.it](mailto:michele.bellesi@unicam.it)

**SUPPLEMENTARY METHODS**

**EEG preprocessing and selection of NREM2 segments**

All EEG preprocessing and analyses were performed in MATLAB using custom scripts together with FieldTrip and EEGLAB (2021). EEG data were band-pass filtered between 0.5 and 35 Hz using zero-phase FIR filtering. Artifacts were identified using an automated epoch-based rejection procedure based on kurtosis and joint probability. Contiguous artifact-free stage 2 NREM (NREM2) segments of at least 5 minutes were selected, and fixed-length 5-minute epochs were extracted for analysis. A frontal–central signal of interest was obtained by averaging the two available bipolar derivations, approximating centro-parietal regions where sigma activity is maximal.

**Sleep staging and quality control**

Sleep staging was derived from automated hypnogram files generated by the portable EEG system. The Dreem algorithm, validated against expert-scored polysomnography, shows good agreement for sleep stage classification (∼83–84% accuracy) [[1]](https://www.zotero.org/google-docs/?nhtGJx). To minimize misclassification, all artifact-free NREM2 segments were visually inspected using a custom interface. Segments with residual artifacts or stage-inconsistent EEG features were excluded. Only NREM2 segments independently validated by two expert scorers were retained for analysis, ensuring reliable infraslow measurements.

**Data post processing and analyses**

**Spindle detection and clustering**

Sleep spindles were detected using the A7 algorithm [[2]](https://www.zotero.org/google-docs/?2j3cCE), an automated detector designed to emulate expert human scoring based on multiple sigma-band features, including relative sigma power, covariance, and signal correlation. The algorithm was applied to EEG signals resampled at 100 Hz, using its standard adaptive framework, and spindles were classified as fast (>12 Hz), and slow (<= 12 Hz). For this analysis we kept exclusively spindle events (N=10340) occurring in the batch of pre-processed, visually inspected 5-min segments of good quality NREM2.

Because the present recordings were acquired in a home setting using a portable device with non-standard bipolar derivations and dry electrodes, the signal characteristics differed from those of conventional laboratory polysomnography datasets on which the detector was originally validated. To account for this, the detector was implemented with slightly more permissive thresholds, and the resulting detections were subsequently inspected to ensure physiological plausibility. This approach allowed us to achieve an acceptable sensitivity.

### **Circular statistics and phase clustering**

To quantify the infraslow modulation of sigma activity, we computed the sigma-band amplitude envelope and filtered it in the 0.01-0.04 Hz range using a zero-phase finite impulse response (FIR) filter with an order of 400. The instantaneous phase of this infraslow component was then obtained (via analytic signal/Hilbert transform framework), yielding a continuous estimate of the sigma-ISO phase.

Each detected spindle was assigned the instantaneous ISO phase at its onset. Phase values were expressed in radians using a cosine convention, where 0 corresponds to the positive peak of the sigma-ISO, *pi* to the negative peak (trough), and ± *pi*/2 to zero crossings.

The distribution of spindle onset phases was analyzed using circular statistics (circstat toolbox matlab). At the pooled level, phase distributions were visualized using circular histograms. Non-uniformity of phase distributions was assessed using the Rayleigh test, which evaluates whether circular data deviate from a uniform distribution [[3]](https://www.zotero.org/google-docs/?9GrHw9). At the subject level, phase clustering was quantified using the resultant vector length (R). R ranges from 0 (uniform distribution) to 1 (perfect phase locking), providing a measure of the strength of spindle clustering around a preferred phase.

**Isolation of ISO**

ISO dynamics were characterized following established procedures described in [[4]](https://www.zotero.org/google-docs/?eqCxWo) and extended in recent human studies [[5]](https://www.zotero.org/google-docs/?c9yB0o). Continuous sigma-band amplitude time courses were derived using time-frequency decomposition, and their power spectra were analyzed in the infraslow range (<0.1 Hz) to quantify rhythmic modulation of sigma activity. Sigma-band activity was estimated using Morlet wavelets (4-cycle wavelets over frequencies from 10 to 16 Hz in 0.2 Hz steps). The amplitude envelope was obtained by computing the absolute value of the complex Fourier coefficients and averaging it across frequencies at 0.1 second steps (10 Hz sampling). Time courses were detrended, demeaned, and smoothed using a moving-average filter (5 s window) to reduce activity unrelated to infraslow dynamics. Power spectral density of the sigma envelope was then computed using Welch’s method (window type: Hanning, 240s with 90% overlap), retaining spectral estimates in the 0-0.12 Hz range. To facilitate comparisons across nights and individuals, spectra were expressed in relative units by normalizing to median infraslow power and averaged across accepted segments within each night.

**ISO feature extraction and quality control**

ISO features were extracted from relative power spectra using a model-based fitting approach. For each spectrum, a three-parameter (frequency, amplitude, width) Gaussian function with an additive constant term (constant baseline) was fitted. This formulation explicitly models the infraslow spectral peak as a localized oscillatory component superimposed on a non-oscillatory spectral background. Although spectra were expressed in relative units to account for variability in overall signal amplitude, retaining a baseline term allows residual broadband structure in the infra-slow range to be estimated directly from the data. This method avoids a priori assumptions about baseline shape (e.g., flat or predefined offsets) and accounts for aperiodic (1/f-like) components of EEG power spectra [[6,7]](https://www.zotero.org/google-docs/?XpfJdN). Fits were restricted to the 0.01-0.04 Hz range, previously shown to capture dominant ISO modulation of sigma power [[8]](https://www.zotero.org/google-docs/?2CJYrs). ISO peak frequency, peak amplitude, and bandwidth were defined from the Gaussian fit as the center frequency, height above baseline, and twice the standard deviation of the oscillatory component, respectively.

To ensure robust detection of meaningful ISO components, spectra were required to exhibit a clear spectral peak exceeding background variability and to show improved fit relative to a constant model. Spectra failing these criteria were excluded from further analyses.

**Power analysis**

EEG spectral power was quantified during NREM2 and NREM3 using pre-processed data segments. Continuous NREM data were divided into non-overlapping 5-s epochs, and power spectra were estimated using a multitaper FFT approach over the 0.5-35 Hz range (using FieldTrip with a multitaper FFT approach with spectral smoothing of 2 Hz, zero-padding to the epoch length, and linear detrending per epoch; frequency range 0.5-35 Hz). Band-limited power was computed for delta (0.5–4 Hz; SWA), alpha (8-12 Hz), sigma (12-16 Hz), and beta (18-35 Hz). Total broadband power was defined as the integrated power over 0.5-35 Hz. Relative power was expressed as the percentage contribution of each band to total broadband power. Epoch-level power estimates were averaged within each sleep stage and night, and subsequently across nights to obtain subject-level mean power values for NREM2 and NREM3. Relative power measures were interpreted as reflecting the spectral composition of NREM activity rather than absolute signal amplitude.

**Supplementary Results (Tables and Figures)**

| ID | Diagnosis | Treatment | Age | Sex | MOCA | GDS | GAD7 | PSQI |
| --- | --- | --- | --- | --- | --- | --- | --- | --- |
| #1 | AD | Memantine 20 mg | 68 | M | 24 | 2 | 1 | 11 |
| #2 | MCI |  | 63 | M | 24 | 5 | 6 | 8 |
| #3 | AD | Rivastigmine 3 mg | 75 | M | 18 | 5 | 11 | 10 |
| #4 | MCI |  | 60 | M | 22 | 3 | 14 | 13 |
| #5 | AD | Memantine 20 mg | 77 | M | 26 | 7 | 3 | 9 |
| #6 | MCI |  | 68 | F | 27 | 5 | 2 | 12 |
| #7 | AD | Memantine 20 mg | 76 | F | 13 | 2 | 4 | 8 |
| #8 | AD | Memantine 20 mg | 81 | M | 28 | 13 | 13 | 11 |
| #9 | MCI |  | 57 | M | 27 | 1 | 1 | 7 |
| *Mean ± std* | | | *69.4 ± 8.3* |  | *23.2 ± 4.9* | *4.8 ± 3.6* | *6.1 ± 5.2* | *9.89 ± 2.0* |
|  | | | | | | | |  |
| #10 |  |  | 76 | M | 29 | 4 | 4 | 13 |
| #11 |  |  | 63 | F | 29 | 0 | 4 | 9 |
| #12 |  |  | 71 | M | 27 | 6 | 10 | 11 |
| #13 |  |  | 66 | M | 28 | 0 | 0 | 8 |
| #14 |  |  | 74 | M | 25 | 3 | 10 | 16 |
| #15 |  |  | 74 | M | 28 | 1 | 1 | 9 |
| #16 |  |  | 63 | M | 29 | 1 | 2 | 9 |
| #17 |  |  | 63 | M | 27 | 4 | 4 | 14 |
| #18 |  |  | 73 | M | 25 | 1 | 0 | 7 |
| #19 |  |  | 77 | F | 25 | 7 | 15 | 19 |
| #20 |  |  | 62 | M | 28 | 0 | 0 | 9 |
| #21 |  |  | 77 | M | 26 | 5 | 9 | 8 |
| #22 |  |  | 60 | M | 26 | 0 | 0 | 9 |
| #23 |  |  | 72 | F | 27 | 1 | 1 | 12 |
| #24 |  |  | 68 | M | 29 | 0 | 0 | 11 |
| #25 |  |  | 70 | M | 25 | 5 | 6 | 17 |
| #26 |  |  | 70 | F | 27 | 1 | 1 | 8 |
| #27 |  |  | 75 | M | 26 | 1 | 1 | 9 |
| *Mean ± std* | | | *69.7 ± 5.6* |  | *27 ± 1.5* | *2.2 ± 2.3* | *3.8 ± 4.5* | *11 ± 3.5* |
| *(AD vs CTR t-test) P=* | | | *0.94* |  | *0.005* | *0.036* | *0.24* | *0.39* |

**Supplementary Table 1. Demographic and clinical characteristics of the study participants.** Values are reported as mean ± standard deviation (SD). Group comparisons (AD vs controls) were performed using two-tailed t-tests. Abbreviations: MoCA, Montreal Cognitive Assessment; GDS, Geriatric Depression Scale; GAD-7, Generalized Anxiety Disorder scale; PSQI, Pittsburgh Sleep Quality Index.

| ID | N. Nights | Sleep time h | %N1 | %N2 | %N3 | % Tot. NREM | % REM | % Wake |
| --- | --- | --- | --- | --- | --- | --- | --- | --- |
| #1 | 6 | 7.3 | 6.2 | 36.2 | 22.8 | 65.2 | 18.9 | 15.9 |
| #2 | 6 | 7.2 | 4.8 | 35.4 | 14.7 | 54.9 | 24.7 | 11.5 |
| #3 | 3 | 9.7 | 14.0 | 50.3 | 7.6 | 71.9 | 9.0 | 15.0 |
| #4 | 2 | 8.0 | 6.6 | 35.3 | 20.7 | 62.7 | 20.6 | 16.7 |
| #5 | 3 | 8.4 | 4.0 | 34.4 | 1.9 | 40.3 | 40.2 | 19.1 |
| #6 | 6 | 7.9 | 5.4 | 47.1 | 18.7 | 71.2 | 8.2 | 20.6 |
| #7 | 1 | 9.3 | 1.9 | 27.6 | 6.2 | 35.7 | 40.8 | 11.4 |
| #8 | 7 | 7.5 | 11.5 | 41.2 | 6.0 | 58.7 | 24.6 | 16.6 |
| #9 | 7 | 7.9 | 7.6 | 53.2 | 17.5 | 78.6 | 16.5 | 4.7 |
|  | 4.6 ± 2.3 | 8.1 ± 0.8 | 6.9 ± 3.8 | 40.1 ± 8.5 | 12.9 ± 7.6 | 59.9 ± 14.4 | 22.6 ± 11.7 | 14.6 ± 4.8 |
|  | | | | | | | |  |
| #10 | 4 | 7.9 | 7.6 | 47.1 | 11.0 | 65.7 | 27.3 | 7.0 |
| #11 | 1 | 8.7 | 3.0 | 67.4 | 13.3 | 83.7 | 2.1 | 14.0 |
| #12 | 5 | 7.6 | 12.6 | 45.9 | 6.3 | 64.9 | 22.1 | 12.8 |
| #13 | 3 | 8.4 | 6.4 | 36.3 | 22.4 | 65.1 | 22.9 | 12.0 |
| #14 | 6 | 9.5 | 6.0 | 30.3 | 28.8 | 65.0 | 10.1 | 24.8 |
| #15 | 6 | 8.3 | 5.4 | 44.9 | 22.1 | 72.4 | 15.2 | 12.4 |
| #16 | 6 | 6.5 | 6.2 | 57.2 | 3.2 | 66.6 | 22.6 | 10.8 |
| #17 | 6 | 8.3 | 7.1 | 35.2 | 14.5 | 56.7 | 21.9 | 21.3 |
| #18 | 1 | 9.0 | 7.2 | 41.7 | 5.3 | 54.1 | 16.3 | 29.6 |
| #19 | 7 | 7.3 | 9.9 | 53.9 | 6.6 | 70.4 | 21.4 | 8.2 |
| #20 | 5 | 7.9 | 4.9 | 49.0 | 13.3 | 67.3 | 9.3 | 23.4 |
| #21 | 5 | 7.4 | 8.9 | 40.2 | 13.1 | 62.2 | 20.3 | 17.5 |
| #22 | 1 | 10.3 | 11.7 | 28.2 | 0.1 | 40.0 | 34.3 | 25.7 |
| #23 | 7 | 8.3 | 5.6 | 42.2 | 15.1 | 62.9 | 26.8 | 10.3 |
| #24 | 7 | 7.2 | 5.0 | 44.4 | 19.3 | 68.8 | 25.0 | 6.3 |
| #25 | 6 | 7.8 | 6.7 | 30.6 | 19.7 | 57.0 | 22.8 | 20.1 |
| #26 | 5 | 7.9 | 6.1 | 44.2 | 11.6 | 62.0 | 31.8 | 6.3 |
| #27 | 2 | 9.3 | 5.9 | 42.0 | 7.0 | 54.8 | 28.8 | 16.3 |
|  | 4.6 ± 2.1 | 8.2 ± 0.9 | 7 ± 2.4 | 43.4 ± 9.8 | 12.9 ± 7.5 | 63.3 ± 9.1 | 21.2 ± 8.1 | 15.5 ± 7.2 |
| Mean ± std | | | 69.3 ± 5.8 |  | 27.1 ± 1.6 | 2.4 ± 2.4 | 4.1 ± 4.7 | 11.3 ± 3.6 |
| (AD vs CTR t-test) P= | | | 0.55 |  | 0.006 | 0.029 | 0.22 | 0.44 |

**Supplementary Table 2**. **Sleep architecture characteristics of the study participants.**

Sleep parameters are reported for each participant and averaged across available recording nights. Values are expressed as mean ± standard deviation (SD). Sleep time is reported in hours. Percentages refer to the proportion of total sleep time spent in each stage (N1, N2, N3, REM) or wake after sleep onset. Total NREM represents the sum of N1, N2, and N3 sleep. Group means (AD and controls) are reported, and between-group comparisons were performed using two-tailed t-tests.

**Supplementary Figure 1. Group-wise data availability for ISO analyses**

**A.** Distribution of the number of nights retained per subject in CTR and AD groups after preprocessing and quality control. **B**. Differences of the average number of valid NREM2 segments per night for each subject. Boxes indicate the interquartile range, central lines the median, whiskers the data range, and dots individual subjects. No significant group differences were observed in the number of nights retained or in the average number of segments per night (all *p* > 0.2).

**Supplementary Figure 2 ISO phase preference for slow and fast spindles.**

A total of n = 10340 spindles were detected and then subdivided into slow (<= 12 Hz, ~54%), and fast (>12 Hz) **A.** Slow spindles. Circular distribution of spindle onset across sigma-ISO phase (left) and subject-level phase clustering (Rayleigh vector length, R) for CTR and AD groups (right). The gray dotted line indicates 10% occurrence probability per bin, and the blue vector denotes the preferred phase. **B.** Fast spindles. Same representation as in (A), showing pooled phase distribution (left) and subject-level phase clustering (right).

**Supplementary Figure 3 Subject-based group differences in ISO features**

Distribution of ISO features in CTR (blue) and AD (yellow). Panels display peak amplitude (A), peak frequency (B), and bandwidth (C) of the ISO. Individual dots represent each subject, half-violins depict kernel density estimates, and horizontal lines indicate the median value within each group.**p < 0.01.

**Supplementary Figure 4 Average power for common frequency bands in CTR and AD in N2 and N3 NREM sleep**

Spectral power was computed from the bipolar fronto–occipital derivation within NREM2 and NREM3 time periods. Power spectra were estimated using a multitaper FFT approach. Relative band power was expressed as a percentage of total power.

**REFERENCES**

[[1]](https://www.zotero.org/google-docs/?PHUGfD)	[Arnal PJ, Thorey V, Debellemaniere E, Ballard ME, Bou Hernandez A, Guillot A, et al. The Dreem Headband compared to polysomnography for electroencephalographic signal acquisition and sleep staging. Sleep 2020;43:zsaa097. https://doi.org/10.1093/sleep/zsaa097.](https://www.zotero.org/google-docs/?PHUGfD)

[[2]](https://www.zotero.org/google-docs/?PHUGfD)	[Lacourse K, Delfrate J, Beaudry J, Peppard P, Warby SC. A sleep spindle detection algorithm that emulates human expert spindle scoring. J Neurosci Methods 2019;316:3–11. https://doi.org/10.1016/j.jneumeth.2018.08.014.](https://www.zotero.org/google-docs/?PHUGfD)

[[3]](https://www.zotero.org/google-docs/?PHUGfD)	[Landler L, Ruxton GD, Malkemper EP. Circular data in biology: advice for effectively implementing statistical procedures. Behav Ecol Sociobiol 2018;72:128. https://doi.org/10.1007/s00265-018-2538-y.](https://www.zotero.org/google-docs/?PHUGfD)

[[4]](https://www.zotero.org/google-docs/?PHUGfD)	[Lecci S, Fernandez LMJ, Weber FD, Cardis R, Chatton J-Y, Born J, et al. Coordinated infraslow neural and cardiac oscillations mark fragility and offline periods in mammalian sleep. Sci Adv 2017;3:e1602026. https://doi.org/10.1126/sciadv.1602026.](https://www.zotero.org/google-docs/?PHUGfD)

[[5]](https://www.zotero.org/google-docs/?PHUGfD)	[Dimitriades ME, Osorio-Forero A, Fattinger S, Arx S von, Kurth S, Pugin F, et al. The Infraslow Fluctuation of Sigma Power During Sleep: Links to Markers of Arousal and Memory Reactivation Across Development 2024:2024.11.06.620875. https://doi.org/10.1101/2024.11.06.620875.](https://www.zotero.org/google-docs/?PHUGfD)

[[6]](https://www.zotero.org/google-docs/?PHUGfD)	[Gerster M, Waterstraat G, Litvak V, Lehnertz K, Schnitzler A, Florin E, et al. Separating Neural Oscillations from Aperiodic 1/f Activity: Challenges and Recommendations. Neuroinformatics 2022;20:991–1012. https://doi.org/10.1007/s12021-022-09581-8.](https://www.zotero.org/google-docs/?PHUGfD)

[[7]](https://www.zotero.org/google-docs/?PHUGfD)	[Donoghue T, Haller M, Peterson EJ, Varma P, Sebastian P, Gao R, et al. Parameterizing neural power spectra into periodic and aperiodic components. Nat Neurosci 2020;23:1655–65. https://doi.org/10.1038/s41593-020-00744-x.](https://www.zotero.org/google-docs/?PHUGfD)

[[8]](https://www.zotero.org/google-docs/?PHUGfD)	[Lecci S, Cataldi J, Betta M, Bernardi G, Heinzer R, Siclari F. Electroencephalographic changes associated with subjective under- and overestimation of sleep duration. Sleep 2020;43:zsaa094. https://doi.org/10.1093/sleep/zsaa094.](https://www.zotero.org/google-docs/?PHUGfD)
